# Microsatellite instability is a biomarker for immune checkpoint inhibitors in endometrial cancer

**DOI:** 10.18632/oncotarget.23790

**Published:** 2017-12-31

**Authors:** Hitomi Yamashita, Kentaro Nakayama, Masako Ishikawa, Kohei Nakamura, Tomoka Ishibashi, Kaori Sanuki, Ruriko Ono, Hiroki Sasamori, Toshiko Minamoto, Kouji Iida, Razia Sultana, Noriyoshi Ishikawa, Satoru Kyo

**Affiliations:** ^1^ Department of Obstetrics and Gynecology, Shimane University School of Medicine, Izumo, Japan; ^2^ Department of Organ Pathology, Shimane University School of Medicine, Izumo, Japan

**Keywords:** microsatellite instability, endometrial cancer, immune checkpoint inhibitor, immunohistochemistry, mismatch repair protein, Immunology

## Abstract

In recent years, it has become evident that tumor cells have immune escape mechanisms, and immune checkpoint inhibitor therapy (anti-PD-1/PD-L1 antibody) has shown benefit in various cancers. In endometrial tumors with microsatellite-instability (MSI), somatic mutations have the potential to encode ‘’non-self’’ immunogenic antigens, and lymphocytes have been shown to infiltrate the tumor. Therefore, immune checkpoint inhibitor therapy might be effective in endometrial cancers with MSI. Expression of mismatch repair (MMR) proteins (MLH1, PMS2, MSH2, and MSH6), the presence of tumor-infiltrating lymphocytes (CD8+), and PD-1/PD-L1 expression were assessed by immunohistochemistry in 149 patients with endometrial cancer. We examined whether tumors with MSI had an enhanced immune microenvironment and whether MSI could be a predictor of the therapeutic effect of PD-1/PD-L1 immunotherapy in endometrial cancer. Loss of MMR protein expression was identified in 42 (28.2%) of 149 patients (MSI group) with endometrial cancer. There was no significant relationship between MSI status and age (*p* = 0.193), histological grade (*p* = 0.097), FIGO stage (*p* = 0.508), pelvic lymph node metastasis (*p* = 0.139), or depth of myometrial invasion (*p* = 0.494). However, the presence of tumor-infiltrating lymphocytes (CD8+) and PD-L1/PD-1 expression were significantly higher in the MSI group compared to the microsatellite-stable group (*p* = 0.002, *p* = 0.001, and p = 0.008, respectively). These results suggest that immune checkpoint inhibitors (anti-PD-1/PD-L1 antibody) could be effective in endometrial cancers with MSI. The presence of MSI may be a biomarker for good response to PD-1/PD-L1 immunotherapy in endometrial cancer.

## INTRODUCTION

Because of the increase in genetic mutation that frequently accompanies tumorigenesis, tumor cells express neoantigens that are different from those on normal cells. Tumor antigens are processed by dendritic cells, and become expressed on the cell surface, together with major histocompatibility complex I and II (MHC class 1 and 2). When CD8 T cells recognize tumor antigens bound to MHC class 1, they become cytotoxic T lymphocytes (CTLs), which can attack cancer cells. However, recent studies have shown that tumor cells have immune escape mechanisms [1, 2]. The programmed cell death-1 (PD-1) and PD-1 ligand 1 (PD-L1) pathway is one such immune escape mechanism. When PD-1 expressed on CTLs binds to PD-L1 expressed on cancer cells, the anti-tumor immune response mediated by CTLs is suppressed. Pharmacologic inhibition of the PD-1/PD-L1 pathway allows for reactivation of the immune response against the tumor [3]. Blockade of this pathway with antibodies to PD-1 and PD-L1 has been reported to be effective in many different types of cancers [4-6]. In addition, it is suggested that immune checkpoint inhibitors may be effective when there is a high infiltration of CD8 lymphocytes into the tumor [16].

Anti-PD-1/PD-L1 antibodies have been used in clinical practice; however, they are not effective in all patients, and response efficiencies when patients of various cancer types are treated by PD-1 antibody are reported to be 20%-30% [6-8]. Moreover, side effects peculiar to immune checkpoint inhibitors (pulmonary toxicity, type 1 diabetes, and colitis) have been reported in patients receiving anti-PD-1/PD-L1 antibodies. Grade 3 or 4 drug-related adverse events occurred in 14% of patients, and, in one study, there were three deaths from pulmonary toxicity [6]. Because of the high drug price and serious reported side effects, discovering biomarkers for immune checkpoint inhibitors is urgent.

It has been reported that, in patients with high PD-L1 expression on tumor cells, PD-1/PD-L1 blockade has a greater therapeutic effect compared to that in patients with low expression of PD-L1 [9-13]. However, it has also been reported that anti-PD-1 antibody is effective even in patients without PD-L1 expression [11]. There are reports that the expression of PD-L1 is dynamic and therefore not suitable as a biomarker. PD-L1 expression on tumor cells in non-small cell lung cancer reveals intratumoral heterogeneity, and can differ between surgical and biopsy specimens. There are no clear criteria for the evaluation of PD-L1 expression, and there are also differences in PD-L1 expression between commonly used assays [14, 15].

In recent years, it has been reported that tumors with higher numbers of somatic mutations (mutation burden rich) are more immunogenic, and immune checkpoint inhibitors are effective for tumors that are mutation burden rich [17-19]. For example, in smokers, the number of genetic mutations in tumors is increased, and immune checkpoint inhibitors have been effective in these patients [19, 20]. Le and colleagues reported significant responses of tumors with microsatellite instability (MSI) to anti-PD-1 antibody in patients who failed conventional therapy [18]. Cancers with MSI have many genetic mutations. High immunogenicity and more immune cell infiltration into the tumor have also been observed in tumors with MSI. Therefore, immune checkpoint inhibitors are thought be effective for tumors with MSI [21].

Endometrial cancer is a gynecological tumor that is frequently MSI positive. Based on genetic features, The Cancer Genome Atlas Research Network provided a new classification system for endometrial carcinoma, which defines four subgroups of cancer (polymerase epsilon (POLE)-ultramutated, MSI-hypermutated, copy number low, and copy number high) [22].

Endometrial cancer is the fifth leading cause of cancer death in the world, and is the most common gynecological cancer [23]. However, advanced endometrial cancer and recurrent endometrial cancer have poor prognosis with current treatments [24-28]. Therefore, we have hypothesized that prognosis may be improved using immune checkpoint inhibitors in endometrial tumors with MSI. In the present study, we investigated biomarkers for predicting the effect of immune checkpoint inhibitors using immunostaining in endometrial carcinoma with MSI.

## RESULTS

### Patients’ clinicopathological characteristics

In the present study, 96 of 149 cases were diagnosed as FIGO stage I, 16 were diagnosed as FIGO stage II, 32 were diagnosed as FIGO stage III, and 5 were diagnosed as FIGO stage IV. The patients were treated initially as follows: total hysterectomy in 59 patients, modified radical hysterectomy in 73, and radical hysterectomy in 13. Four patients underwent chemotherapy and/or radiotherapy without surgery due to complications. Retroperitoneal lymph node dissection was performed in 124 patients. Radiotherapy (whole pelvic irradiation) and/or chemotherapy (paclitaxel 175 mg/m^2^ and carboplatin area under the curve = 5 mg/m^2^) was performed postoperatively in patients with high recurrence risk (deep myometrial invasion, grade 2 or 3; lymph node metastasis; or lymphovascular space invasion). A summary of the clinicopathological characteristics of the patients is provided in Table 1.

**Table 1:** Characteristics of endometrial cancer patients

Characteristic	MSI (+)	MSI (-)	*p-Value*
	***N*** = 42	***N*** = 107	
Age-no. (%)			0.193
<60	25(60)	51(48)	
≥ 60	17(40)	56(52)	
Grade-no. (%)			0.097
G1	18(43)	62(58)	
G2, 3	24(57)	45(42)	
FIGO Stage-no. (%)			0.508
I, II	30(71)	82(77)	
III, IV	12(29)	25(23)	
Pelvic lymph metastasis-no. (%)			0.139
No	28(80)	80(90)	
Yes	7(20)	9(10)	
Muscle invasion-no. (%)			0.494
< 50%	25(61)	69(67)	
≥ 50%	16(39)	34(33)	

**Figure 1:**
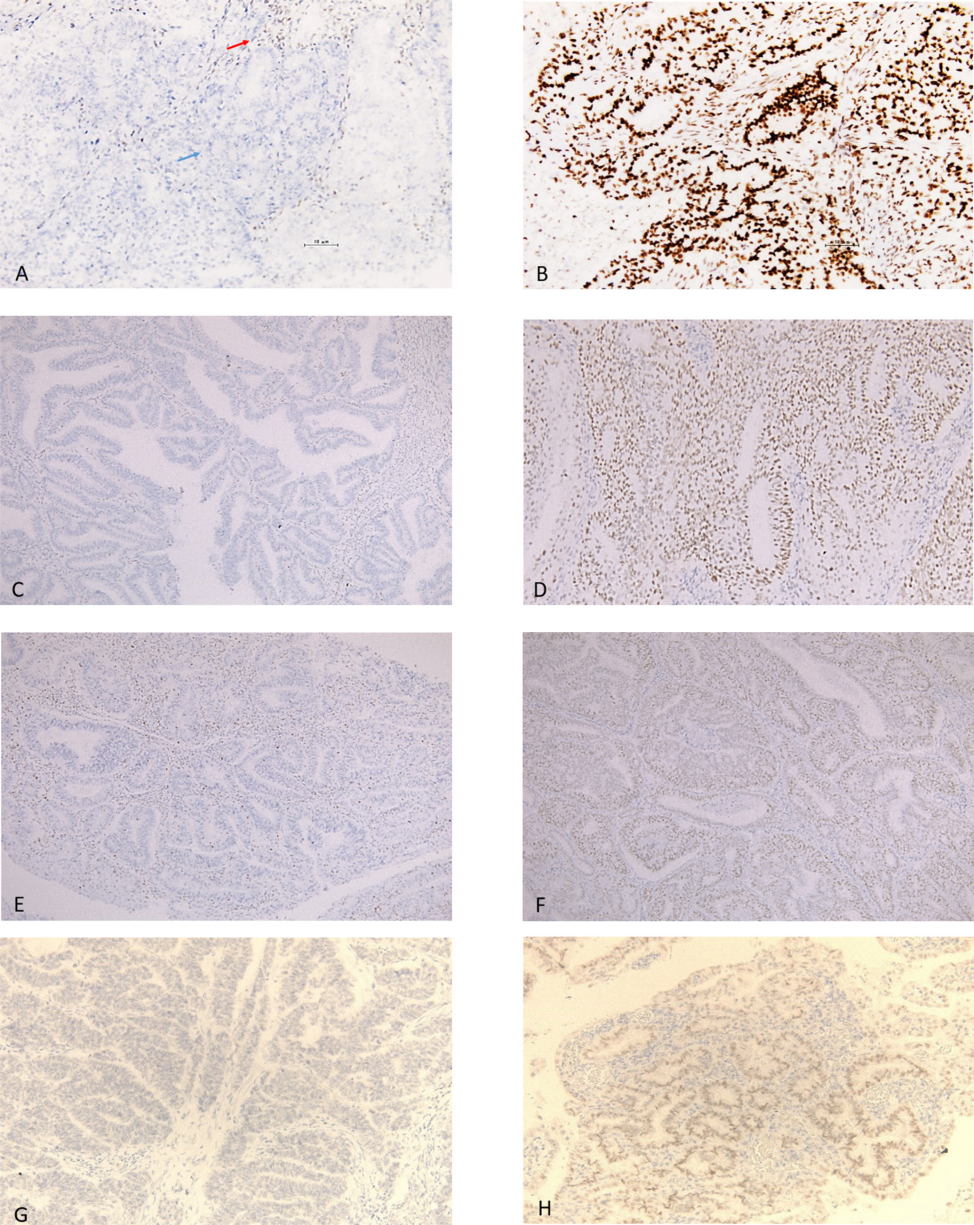
Immunostaining of mismatch repair proteins **A.** Loss of expression of MLH1. The immunostaining is positive in stromal cells (red arrow) and negative in tumor cells (blue arrow). **B.** Expression of MLH1. **C.** Loss of expression of MSH2. **D.** Expression of MSH2. **E.** Loss of expression of MSH6. **F.** Expression of MSH6. **G**. Loss of expression of PMS2. **H**. Expression of PMS2.

In the present study, 42/149 patients were MSI-positive (28.2%) (MSH2 loss, 21 cases; MSH6 loss, 14 cases; MLH1 loss, 20 cases; and PMS2 loss, 3 cases). Figure 1 shows representative cases that were positive and negative for MLH1, MSH2, MSH6, and PMS2. There was no significant relationship between MSI status and age (*p* = 0.193), histological grade (*p* = 0.097), FIGO stage (*p* = 0.508), pelvic lymph metastasis (*p* = 0.139), or depth of myometrial invasion (*p* = 0.494) (Table 1).

### Relationships between MSI and CD8, PD-L1, and PD-1 expression

**Figure 2:**
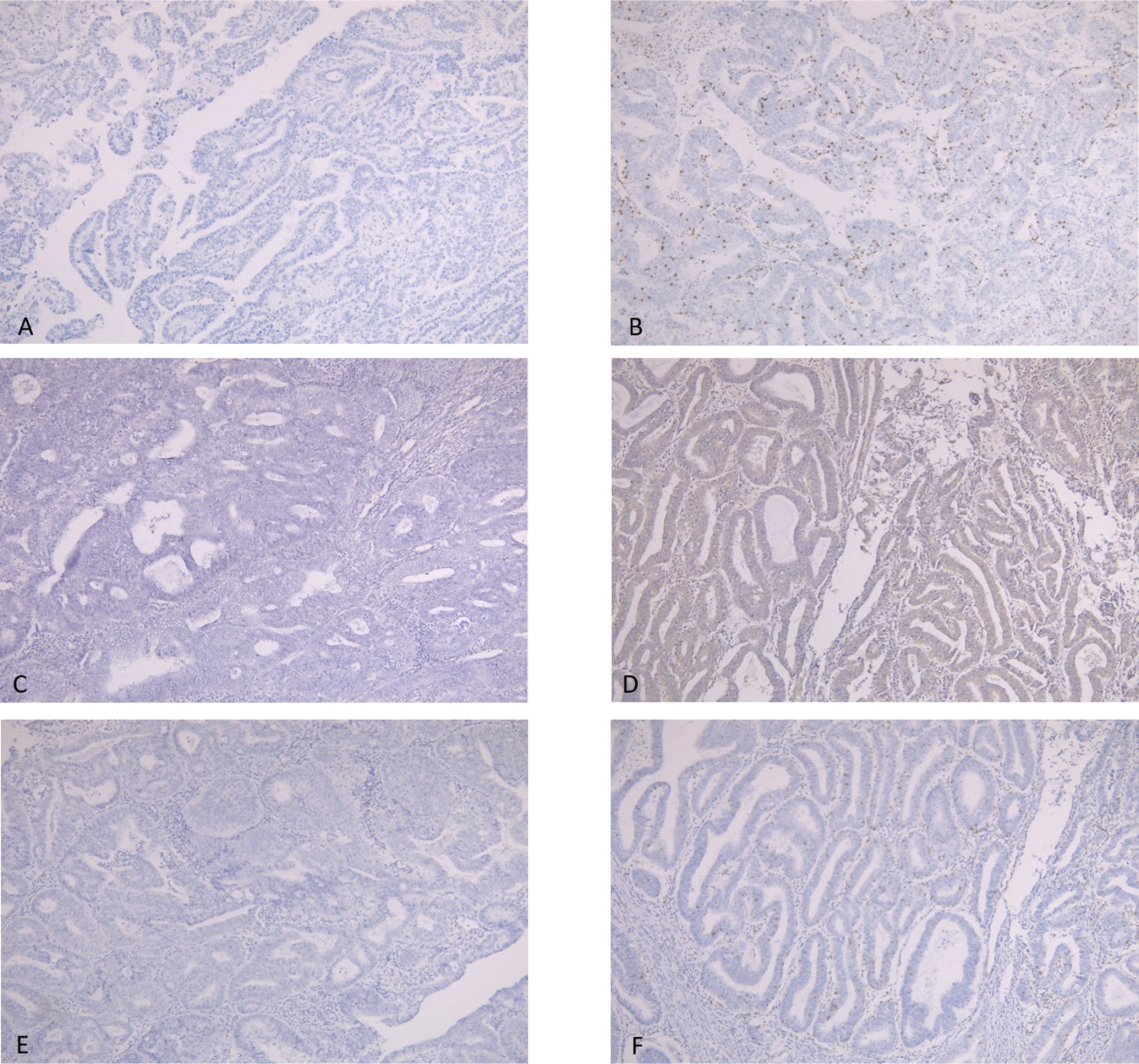
**A.,** **B.**, Immunostaining of CD8. A, CD8 expression score of 0. B, CD8 expression score of 3+. **C.**, **D.** Immunostaining of PD-L1. C, no expression of PD-L1. D, positive expression of PD-L1. **E.**, **F.** Immunostaining of PD-1: E, no expression of PD-1. F, positive expression of PD-1.

The relationships between MSI and the expression of CD8, PD-L1, and PD-1 were assessed using a Chi-squared test. The positive rate of CD8 expression in MSI cases was higher than that in MSS cases (p = 0.002) (Table 2, Figure 2A-2B). Similarly, the expression rates of PD-L1 and PD-1 were higher in MSI cases than in MSS cases (*p* = 0.008, *p* = 0.001, respectively) (Tables 3 and 4, Figure 2C-2F).

**Table 2:** Relationship between status of MSI and CD8 expression

Parameter	MSI (+)	MSI (-)	*p-Value*
	*N* = 42	*N* = 107	
CD8-no. (%)			0.002
positive	23(55)	30(28)	
negative	19(45)	77(72)	

**Table 3:** Relationship between status of MSI and PD-L1 expression

Parameter	MSI (+)	MSI (-)	*p-Value*
	*N* = 42	*N* = 107	
PD-L1-no. (%)			0.008
positive	20(48)	27(25)	
negative	22(52)	80(75)	

**Table 4:** Relationship between status of MSI and PD-1 expression

Parameter	MSI (+)	MS I (-)	*p-Value*
	*N* = 42	*N* = 107	
PD-1-no. (%)			0.001
positive	12(29)	8(7)	
negative	30(71)	99(93)	

### MSI analysis

**Figure 3:**
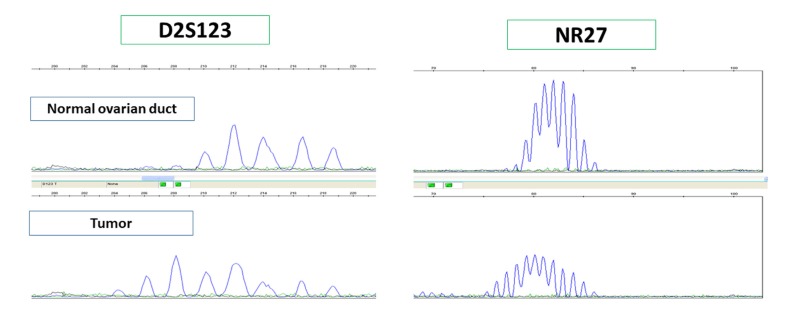
MSI analysis was performed in cases that were negative for one or more MMR protein by immunostaining to confirm MSI

We conducted genomic microsatellite instability analysis in 12 cases evaluated as MSI by IHC. All cases were diagnosed as MSI by microsatellite analysis; therefore, we believe that MSI assessment by immunostaining was valid (Figure 3).

### Univariate analysis of prognostic factors in patients with endometrial carcinoma

**Figure 4:**
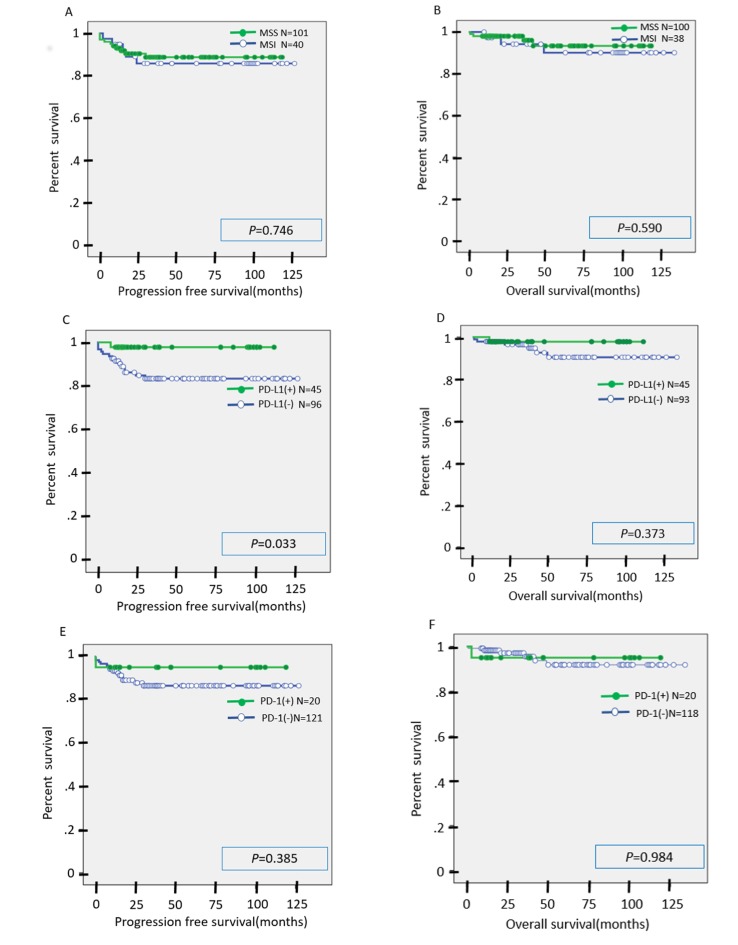

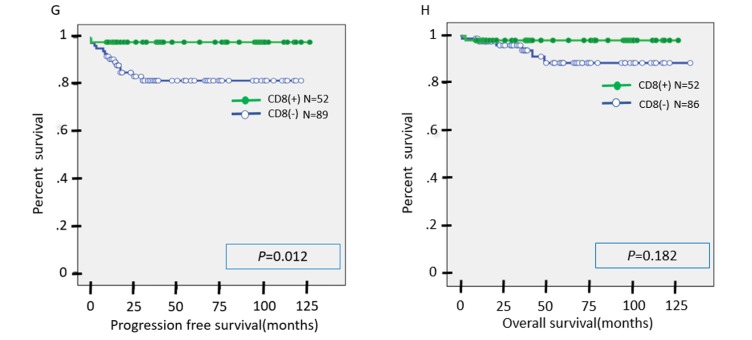
**A.,** **B.** Kaplan-Meier analysis of progression-free (A) and overall (B) survival between the MSI group and MSS group. **C.**, **D.** Kaplan-Meier analysis of progression-free (C) and overall (D) survival between the PD-L1(+) group and PD-L1(-) group. **E.**, **F.** Kaplan-Meier analysis of progression-free (E) and overall (F) survival between the PD-1(+) group and PD-1(-) group. **G.**, **H.** Kaplan-Meier analysis of progression-free (G) and overall (H) survival between the CD8(+) group and CD8(-) group.

In the univariate analysis, there was no significant difference in PFS or OS between the MSI group and the MSS group (Figure 4A-4B). Similarly, there was no significant difference in OS between the PD-L1(+) and PD-L1(-) cases, although PFS was significantly prolonged in the PD-L1(+) cases compared to the PD-L1(-) cases (Figure 4C-4D). There was no significant difference in PFS or OS between the PD-1(+) and PD-1(-) cases (Figure 4E-4F). There was no significant difference in OS between the CD8(+) and CD8(-) cases, but PFS was significantly prolonged in CD8(+) cases as compared to CD8(-) cases (Figure 4G-4H). We suspect that second or third line chemotherapies may contribute to overall survival after the first recurrence. This may explain why there was no significant difference in OS between CD8(+) and CD8(-) cases or between PD-L1(+) and PD-L1(-) cases.

## DISCUSSION

Seventy-five percent of women diagnosed with endometrial cancer have early stage disease (stage I or II) and favorable outcomes (5-year overall survival, 75-90%) [32-34]. However, patients who are diagnosed with advanced disease and patients with recurrent disease have poor prognosis when receiving conventional chemotherapy [35, 36]. Therefore, we consider that the personalization of patient care is important.

In previous reports, the ratio of MSI in endometrial carcinoma is 15-30% [37-39]. In the present study, 42/149 (28.2%) patients were MSI-positive. There are different techniques for the identification of MMR deficiency [40-45]. Primarily, MSI analysis and IHC of MMR proteins are used to determine whether tumors are MSI-positive. MSI analysis has primarily been utilized in research, not clinical practice, and requires DNA extraction from the tumor as well as normal tissue or blood for comparison. High concordance between IHC of MMR proteins and MSI has been reported [41]. Immunostaining of MMR proteins is simple and can be performed at any hospital. Moreover, it is more cost effective than MSI analysis. Therefore, we believe that IHC of MMR proteins should be used clinically to identify MSI-positive tumors. In MSI tumors, most cases are MLH1 or MSH2 deficient, but cases of PMS2 and MSH6 deficiency are also seen. Therefore, immunostaining of PMS2 and MSH6 is useful for MSI screening [46].

**Figure 5:**
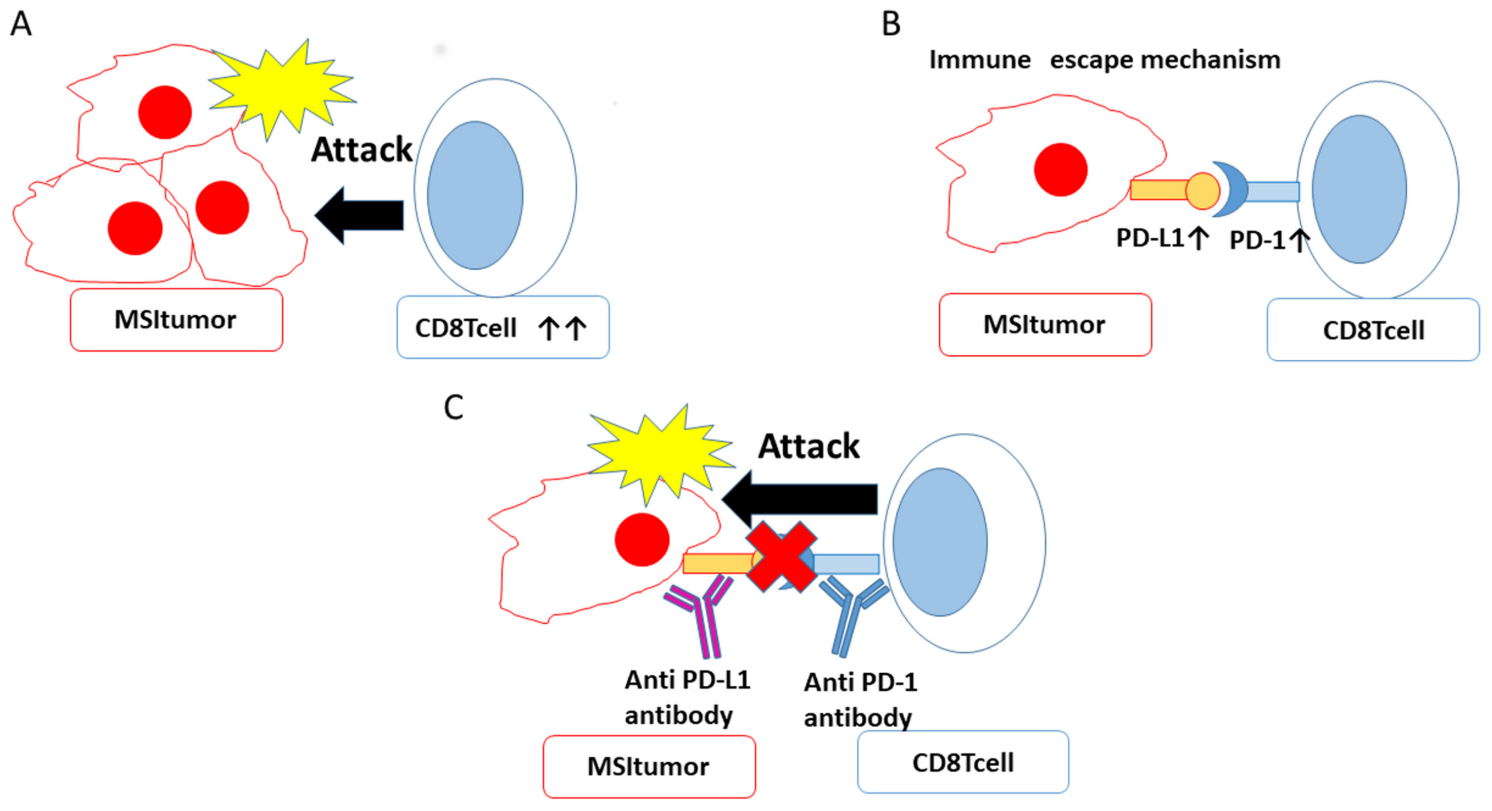
MSI allows tumor cells to escape the immune response and can be targeted by immune checkpoint inhibitors **A.** CD8 lymphocytes initially attack tumors cells. **B.** When PD-L1 on tumor cells binds PD-1 on immune cells, the immune escape mechanism is activated. **C.** Treatment with PD-1 and PD-L1 inhibitors can block this immune escape mechanism.

In the present study, more MSI cases than MSS cases showed CD8 positivity. Although lymphocyte infiltration is a good prognostic factor, there was no significant difference in OS between MSI cases and MSS cases in the current study. However, we also performed univariate analysis of PFS and OS separately in stage I/II and stage III/IV cases, and found that, in stage I/II cases, the MSI group had significantly shortened PFS compared to the MSS group. There was no significant difference between MSI and MSS in OS when stratifying by stage (Supplementary Figure 1, 2). There have been reports indicating that MSI is a good prognostic factor in colorectal cancer and that MMR status does not contribute to prognosis in colorectal cancer [47, 48, 49]. There are many reports that there is no significant difference in survival rate between the MSI and MSS groups in endometrial cancer [50, 51, 52, 53]. Taken together with the current results, the effect of MSI status on prognosis remains controversial. In the current study, as with CD8 expression, more MSI cases than MSS cases were positive for PD-1 and PD-L1. We hypothesize that, in MSI tumors, CD8 lymphocytes initially attack tumor cells, but the immune escape mechanism eventually works by raising PD-L1 expression in tumor cells and PD-1 expression in lymphocytes. Therefore, tumor cells escape attack from immune cells and the tumor can progress (Figure 5). The immune escape mechanism functions in MSI tumors. Therefore, immune checkpoint inhibitors (anti-PD-1, anti-PD-L1 antibodies) may be more effective in MSI cases than in MSS cases. The relationship between MSI and prognosis is thought to vary depending on the extent to which the immune escape mechanism is active. In this study, the group with high PD-L1 expression had significantly prolonged PFS. The relationship between PD-L1 expression and prognosis has been studied in multiple cancer types. There are reports that PD-L1 expression is a good prognostic factor and, conversely, reports that PD-L1 expression is a poor prognostic factor [54, 55, 56, 57]. Our hypothesis is that if PD-L1 expression is increased in cancer cells, the immune escape mechanism is activated in the tumor microenvironment, leading to poor prognosis. Since immune cells infiltrate the tumors before PD-L1 expression increases in MSI tumors, we speculate that patients with PD-L1 expression have a good short-term prognosis due to the influence of CD8 lymphocytes, which are a good prognostic factor. Evaluation of MSI in endometrial cancer is useful for determining whether a patient will respond to immune checkpoint therapy. While we were preparing this manuscript, there was a report that MSI-H endometrial tumors have increased immune cell infiltration and PD-L1 expression compared with MSS endometrial tumors [58]. We consider that their result supports our results.

Mutation burden is expected to be a biomarker for immune checkpoint inhibitors. It has been suggested that not only the number of neoantigens, but also the clonality of genetic abnormalities, may be important for predicting the therapeutic effect of immune checkpoint inhibitors [59]. Moreover, previous studies have found that genetic abnormalities are heterogeneous, even within the same tumor, and it is obvious that neoantigens clonally expressed in the tumor are infiltrated by CD8 positive T cells that have high expression of PD-1 [59, 60]. It has been reported that immune checkpoint inhibitors have more efficacy in cases with many clonal genetic abnormalities and mutation burden rich cases [59, 60]. In endometrial cancers that are mutation burden rich because of POLE mutations, the immune checkpoint molecules are highly expressed, suggesting the possibility that immune checkpoint inhibitor treatment may be effective [61]. Currently, we are investigating whether immune checkpoint inhibitors are also effective for Japanese endometrial cancer patients with POLE mutations.

Not only positive predictive factors for immune checkpoint inhibitors, but also negative biomarkers, have been reported. Loss of PTEN and inactivation of the IFN-γ/JAK/STAT pathway have been reported as negative biomarkers for response to immune checkpoint inhibitors [62-64]. Work is now ongoing to evaluate whether these factors are also negative biomarkers for immune checkpoint inhibitors in endometrial carcinoma. In addition to PD1/PD-L1, CTLA4 has recently received attention as an immune checkpoint molecule. CTLA4 is expressed in activated T cells and regulatory T cells and has an immunosuppressive function. Anti-CTLA4 antibody has been reported to prolong survival in progressive melanoma and was approved by the FDA as an immune checkpoint inhibitor against progressive melanoma in 2011 [65]. We are currently investigating whether anti-CTLA4 antibody is also effective in endometrial cancer.

In summary, various biomarkers of response to immune checkpoint inhibitors have been reported. In this study, PD-1 and PD-L1 expression were significantly higher by immunostaining in MSI cases of endometrial carcinoma as compared to MSS cases. In May 2017, the Food and Drug Administration approved the use of the PD-1 antibody pembrolizumab for solid cancers with MSI-H or MMR-deficiency [66]. Our data support the idea that immune checkpoint inhibitors could be effective in MSI cases of endometrial cancer, even in Japanese populations; however, this should be directly tested, preferably in a large cohort, prospective study. The presence or absence of MSI by immunostaining may be a biomarker for immune checkpoint inhibitors.

## MATERIALS AND METHODS

### Ethics statement

This investigation was conducted in accordance with the ethical standards and according to the Declaration of Helsinki and national and international guidelines, and has been approved by the institutional review board of Shimane University Hospital. Tumor specimens were collected after obtaining written consent from all patients with the approval of the Facility Ethical Committee (Shimane University Hospital; approval no. 2004-0381).

### Tissue samples

Samples were collected from 149 patients with endometrioid-type endometrial carcinomas treated between January 2006 and January 2017 in the Department of Obstetrics and Gynecology at the Shimane University Hospital. The samples were formalin-fixed and paraffin-embedded tissue blocks. The tumors were diagnosed based on conventional morphological examination of hematoxylin and eosin-stained sections.

Endometrial carcinomas were classified according to the surgical staging system of the International Federation of Gynecology and Obstetrics (FIGO 2008) [29]. All tumors were classified histologically according to the World Health Organization criteria.

The relevant clinical data were collected by retrospective review of the patients’ files. The follow-up period ranged from 1 month to 134 months, with a median follow-up of 38 months.

### Immunohistochemistry

Expression of mismatch repair (MMR) proteins (MLH1, PMS2, MSH2, and MSH6), CD8, PD-L1, and PD-1 was evaluated by immunohistochemistry (IHC).

Formalin-fixed and paraffin-embedded sections (4-µm thick) were dewaxed in xylenes and hydrated in graded alcohol. After antigen retrieval in a sodium citrate buffer, slides were incubated overnight at 4°C with antibodies against MutS Protein Homolog 2 (1:50; Dako, Santa Clara, CA, United States), MutS Protein Homolog 6 (1:50; Dako), Postmeiotic Segregation Increased 2 (1:40; Dako), MutL Protein Homolog 1 (1:50; Dako), CD-8 (1:100; Roche, Basel, Switzerland), PD-L1 (ab205921, Abcam, Cambridge, United Kingdom), and PD-1 (Roche). Two researchers who were blinded to the clinicopathological factors evaluated the samples by a light microscope.

### Definition of MSI and CD8/PD-1/ PD-L1 positivity by IHC

Tumors were considered MSI if at least one of the four MMR proteins (MSH2, MSH6, PMS2, or MLH1) was negative.

The population density of tumor infiltrating lymphocytes was stratified into four categories by CD8 staining: 0, undetectable; 1+, low density (0-30%); 2+, moderate density (30-60%); and 3+, high density (≥ 60%). Cases that were 2+ or 3+ were counted as positive in our analysis. For PD-L1, tumors with staining in ≥ 5% of the tumor cells (membranous and cytoplasmic staining) were considered positive. For PD-1, cases with staining in ≥ 5% of the tumor infiltrating lymphocytes were considered positive.

### DNA extraction and microsatellite instability analysis

To validate the evaluation of MSI by immunostaining, we conducted microsatellite instability analysis in 12 cases evaluated as MSI by immunostaining. We digested tumor tissues (0.01 M NaCl; 0.5 M Tris-HCl, pH 8.0; 20 mM EDTA; 0.05% Tween-20; 0.1 mg/mL proteinase K) overnight at 58°C. To inactivate the proteinase K, we heated the tissues to 95°C for 10 min. After that, DNA was extracted with phenol/chloroform treatment and ethanol precipitation. We performed MSI analysis using the polymerase chain reaction (PCR) with eight microsatellite markers (BAT25, BAT26, D2S123, D5S346, D17S250, NR21, MONO27, and NR2). PCR was performed in a total volume of 10 μL containing 25-50 ng of DNA, each sense primer, 20 μM dNTPs, 0.4 μM of each primer, and 0.25 units of TKs Gflex DNA polymerase (Takara Shuzo, Shiga, Japan). We heated the mixtures at 94°C for 10 min, and PCR was performed for 45 cycles at 94°C for 30 s, at the appropriate annealing temperature for 30 s, and at 72°C for 1 min each, followed by 72°C for 10 min [30].

We analyzed the amplicons on the ABI PRISM 310 Genetic Analyzer and evaluated allelic sizes by GeneMapper (Applied Biosystems, Thermo Fisher K. K, Yokohama, Japan). These markers include the recommended markers for the detection of MSI proposed at the National Cancer Institute collaborative meeting on MSI in colorectal carcinoma [31]. When two or more markers showed length variation between the fallopian tube samples (used as normal tissue controls) and the tumor samples, we judged the cases to be MSI. When none of the markers showed length variations between the fallopian tube samples and the tumor samples, we judged the cases to be MSS.

### Statistical analyses

Chi-squared tests were used to analyze the association between MSI and the expression of CD8, PD-1, and PD-L1. Univariate analysis was performed using binomial logistic regression for ordered categorical variables. The endpoints of the analysis were progression-free survival (PFS) and overall survival (OS). PFS and OS were calculated between the date of diagnosis and the date of first relapse and last follow-up, respectively. Because 11 patients were lost to follow-up due to transfer, for univariate analysis we analyzed PFS in 141 patients and OS in 138 patients. Data were plotted as Kaplan-Meier curves, and statistical significance was determined using the log-rank test. In the present study, p-values below 0.05 were considered statistically significant.

## SUPPLEMENTARY MATERIALS FIGURES



## Abbreviations

FIGO: International Federation of Gynecology and Obstetrics
IHC: immunohistochemistry
MSI: microsatellite instability
MMR: mismatch repair
OS: overall survival
PCR: polymerase chain reaction
PD-1: programmed cell death-1
PD-L1: programmed cell death-ligand 1
PFS: progression-free survival
