# Supplementary material for: Microsatellite instability is a biomarker for immune checkpoint inhibitors in endometrial cancer
Source: Oncotarget. 2017 Dec 31;9(5):5652–64. doi: 10.18632/oncotarget.23790 (PMC5814165; doi:10.18632/oncotarget.23790)
Supplement: Supplementary file 1 [file oncotarget-09-5652-s001.pdf]

Microsatellite instability is a biomarker for immune checkpoint inhibitors in endometrial cancer

SUPPLEMENTARY MATERIALS

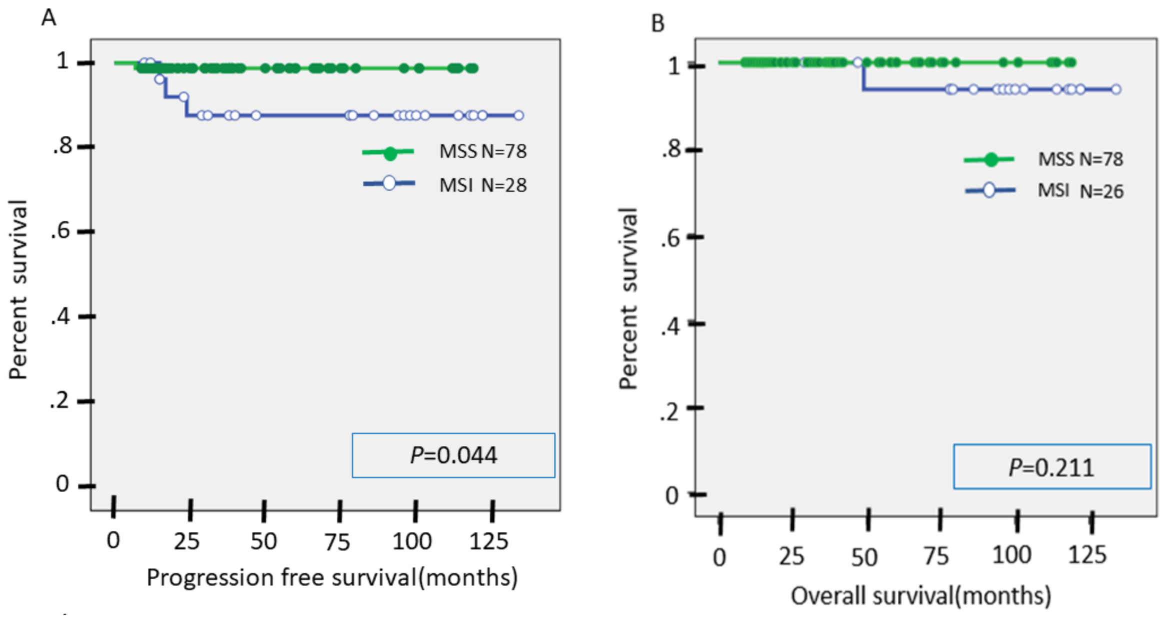

Supplementary Figure 1: A, B Kaplan-Meier analysis of progression-free (A) and overall (B) survival between the MSI group and MSS group in stage I/II cases.

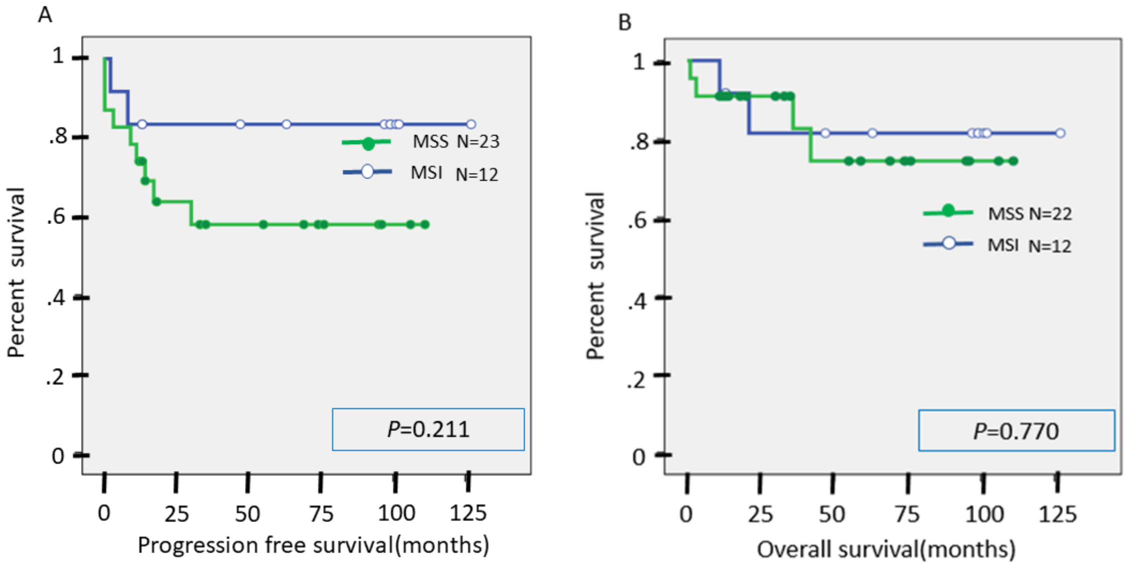

Supplementary Figure 2: A, B Kaplan-Meier analysis of progression-free (A) and overall (B) survival between the MSI group and MSS group in stage III/IV cases.
